# Recommendations for refining key maternal health policy and finance indicators to strengthen a framework for monitoring the Strategies toward Ending Preventable Maternal Mortality (EPMM)

**DOI:** 10.7189/jogh.11.02004

**Published:** 2021-10-23

**Authors:** R Rima Jolivet, Jewel Gausman, Ana Langer

**Affiliations:** Improving Maternal Health Measurement Capacity & Use Project; Women & Health Initiative; Department of Global Health & Population; Harvard T.H. Chan School of Public Health; Boston, Massachusetts, USA)

The Millennium Development Goals (MDGs) comprised 8 goals, 18 targets, and 48 indicators; the Sustainable Development Goal (SDG) framework includes 17 goals, 169 targets, and 232 indicators. Similar expansion is seen in maternal newborn health (MNH) measurement [1]. In a 2019 systematic review, 1445 unique MNH indicators were identified [2]. The burden associated with proliferating indicators necessitates greater focus on quality for those chosen for monitoring.

Various sources propose criteria for evaluating measure quality, which generally include importance, relevance, utility, validity, feasibility, and distinctiveness [3-5]. How clearly the indicator definition captures the underlying construct for measurement, and whether its metadata are constructed so it is reliable, valid, and interpretable [2,6], further influence indicator quality. Effectiveness, the degree to which any objective is achieved [7], can only be evaluated when objectives are: a) clearly defined a priori and b) quantifiable [8]. Poorly defined measures are thus unlikely to be valid, or effective in tracking changes, with the ultimate goal of driving improvement. Therefore, it is concerning that many MNH measures in use lack scientific soundness as evidenced by a complete definition; demonstrated validity, reliability, and feasibility; and plausible application [2].

Health policy indicators are systematically evaluated less frequently than clinical indicators, despite growing awareness of the need for robust data to drive health policy decision-making, system improvement, and accountability to address upstream determinants of maternal health and survival. Efforts to assess validity of indicators designed for monitoring health system and policy factors are challenged by a lack of systematic approaches [9].

Indicators to monitor maternal health financing present particular measurement challenges. A major focus of the SDGs is closing financing gaps to achieve a “grand convergence” [10] in MNH outcomes between countries across income levels, through adequate domestic resource allocation and harmonization in Official Development Assistance (ODA). The Global Financing Facility (GFF) was established in 2015 to reform RMNCH financing to meet the SDGs, and now includes 36 countries [11-13]. Universal Health Coverage (UHC), with prioritization of MCH, is considered key to achieving SDG 3.1 [14]. The former UN Secretary General noted achieving UHC depends upon effective measures for tracking health financing and spending, both governmental and out-of-pocket [15]. However, most health financing measures are not optimized for MNH monitoring. Measurement experts have reported interest in MNH health finance indicators [9]; however, many common economic indicators are not disaggregated to allow tracking of maternal health financing, not routinely collected or reported, or not available in the public domain, among other issues [16-19].

In 2015, WHO released the Strategies toward Ending Preventable Maternal Mortality (EPMM) (EPMM Strategies) [20] to serve as a global framework for maternal health during the SDGs. Subsequently, indicators tailored to the report’s 11 Key Themes [20] were identified for a monitoring framework comprising: a core set maternal health indicators for global reporting [21], and a menu of indicators for national monitoring to track a broad range of social, political, economic and health system determinants of maternal health and survival [22]. The latter were selected through a five-round modified Delphi process to identify the 1-3 strongest available measures to monitor progress toward each EPMM Key Theme. The selection criteria utilized in this process appear in **Table 1**.

**Table 1:** EPMM indicator selection criteria

**Relevance**
• Indicator directly supports EPMM Strategies for reducing preventable maternal mortality
• There is evidence that what the indicator measures is significantly associated with improved maternal health and survival
**Importance**
• Indicator resonates, and is valuable to decision makers and stakeholders
• Indicator “makes a difference” for improving maternal health and survival across countries and contexts
**Interpretability and usefulness**
• There is good/strong evidence to support the process, or the outcome
• Results point to areas for improvement and can advance strategic planning, policy or programming at different levels of the system
**Validity**
• Indicator measures what it is supposed to measure
• Indicator has been field-tested and used
• Indicator makes sense logically and scientifically
**Feasibility and data availability**
• Based on the best available data of acceptable quality
• Data can be obtained with reasonable and affordable efforts in timely manner
• Data does not overly increase reporting burden on countries
**Harmonization**
• Indicator strengthens or compliments existing efforts
• Indicator is recommended and being used by leading experts and organizations
• Indicator lacks redundancy and does not measure something already captured under other indicators

In 2017, the Women and Health Initiative (W&HI) at the Harvard T.H. Chan School of Public Health initiated the Improving Maternal Health Measurement (IMHM) Project, whose primary aim is to strengthen indicators for monitoring the EPMM Strategies. In December 2018, the W&HI convened technical experts in maternal health policy (Consultation 1) and maternal health financing (Consultation 2) to address problems in a selection of these measures. The specific aim was a set of recommendations to improve the validity and utility of selected measures for monitoring key themes of the EPMM Strategies. As many EPMM indicators bridge domains, a secondary aim was intersectoral coordination to improve measurement capacity overall. This paper summarizes the recommendations that emanated from these deliberations.

## Participants

We purposively invited experts in MNH measure development and the topical areas covered by the selected indicators. We specifically included an expert affiliated with the data custodian agency whenever possible, and measurement experts with experience implementing each indicator. In total, forty participants from thirteen countries (Ghana, USA, Brazil, UK, Argentina, Switzerland, Kenya, Bangladesh, Nigeria, Germany, Belgium, Congo-Brazzaville, and India) attended two consecutive domain-specific technical consultations; some participants attended both. There were twenty-six participants in Consultation 1 and twenty-seven in Consultation 2 (Appendix S1 of the **Online Supplementary Document**).

## Conference structure

### Selection of indicators for strengthening

Five maternal health policy indicators (Consultation 1) and five maternal health financing indicators (Consultation 2) were included, presented with full metadata in **Table 2**. From 2017-2018, stakeholders were queried regarding EPMM indicators in need of strengthening through the IMHM Project, during a review of EPMM indicator use in 20 countries, a global stakeholder meeting to prioritize EPMM indicators for validation research, and a poll of IMHM Project advisors. Problems with eighteen EPMM indicators were mentioned in forty-seven instances. The identified indicators were grouped into three domains: maternal health policy, financing, and service delivery. The final selection of indicators was made with inputs from global advisors to ensure harmonization of efforts.

**Table 2:** Ten EPMM indicators for refinement with full metadata

Indicator	Definition	Calculation	Disaggregation	Data source	Indicator reference
**Five maternal health policy indicators**					
1. Legal status of abortion	Definition: The legal grounds under which abortion is allowed. Criteria for ranking: I = to save a woman’s life II = to preserve physical health and above III = to preserve mental health and above IV = for economic and social reason and the above V = on request and above R = in case of rape or incest F = in case of fetal impairment — = data are not available	☐ Yes ☐ No	N/A	Country laws/policies	Countdown to 2030
2. Is there a national policy to ensure engagement of civil society organization representatives in periodic review of national programs for RMNCAH?	Is there a national policy to ensure engagement of civil society organization representatives in periodic review of national programs for RMNCAH	☐ Yes ☐ No ☐ Unknown	N/A	Self-reported by government and UN country offices via survey	WHO Global Reproductive, Maternal, Newborn, Child, and Adolescent Health (RMNCAH) Policy Survey (2018)

3. Presence of a national set of indicators with targets and annual reporting to inform annual health sector reviews and other planning cycles	Indicators cover key issues including health determinants, health system inputs, processes and outputs, use of health care services, mortality, morbidity, health system responsiveness, etc.	☐ Yes ☐ No	N/A	National health sector reports	WHO Health Information Systems Performance Index (2010)
4. Number of countries with laws and regulations that guarantee full and equal access to women and men aged 15 years and older to sexual and reproductive health care, information and education	13 specific components of sexual and reproductive health care, information and education as follows:	SDG 5.6.2. Metadata	By section and component	Self-reported by government with UN country offices via survey	SDG 5.6.2. Survey Tool
	Section I: Maternity Care Services 1. Maternity care 2. Life-saving commodities 3. Abortion 4. Post-abortion care				
	Section II: Contraception and Family Planning 5. Contraception 6. Consent for contraceptive services 7. Emergency contraception				
	Section III: Comprehensive Sexuality Education and Information 8. CSE law 9. CSE curriculum				
	Section IV: Sexual Health and Well-Being 10. HIV testing and counselling 11. HIV treatment and care 12. Confidentiality of health status for men & women living with HIV 13. HPV vaccine				
5. Proportion of women aged 15-49 y who make their own informed decisions regarding sexual relations, contraceptive use and reproductive health care	Proportion of women aged 15-49 y (married or in union) who make their own decision on all three selected areas: 1. Who usually makes decisions about health care for yourself? 2. Who usually makes the decision on whether or not you should use contraception?	Questions 1 & 2: ☐ Respondent ☐Husband/Partner ☐ Respondent and Husband/Partner Jointly ☐ Someone else ☐ Other/Specify	By age, geographic location, place of residence, education, and wealth quintile	Nationally representative household surveys: DHS; MICS; GGS; and other country- specific	SDG 5.6.1.
	3. Can you say no to your husband/partner if you do not want to have sexual intercourse?	Question 3: ☐ Yes ☐ No ☐ Depends/Not Sure			
	*Only women who answer “Yes” to all three questions are included in the numerator.				

CSE – comprehensive sexuality education, DHS – Demographic and Health Surveys, EPMM – Ending Preventable Maternal Mortality, GGS – Generations and Gender Surveys, HIV – human immunodeficiency virus, HPV – human papilloma virus, MICS – Multiple Indicator Cluster Surveys, RMNCAH – Reproductive, Maternal, Newborn, Child, and Adolescent Health, SDG – Sustainable Development Goals, WHO – World Health Organization

**Figure:**
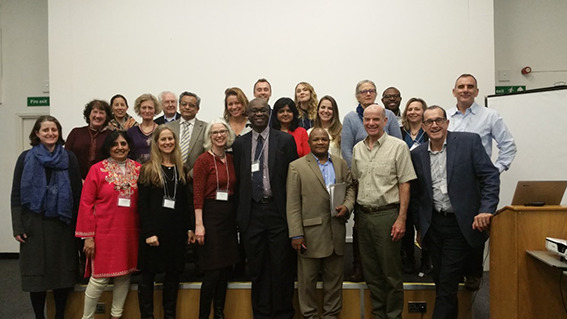
Photo: Women & Health Initiative, Harvard T.H. Chan School, 2018 (from the author’s own collection, used with permission).

### Consultation process

The full metadata (standard indicator name, definition, numerator, denominator, calculation, disaggregation, and data sources) were presented with an overview of data generated from the indicator across geographies and time. Speakers shared perspectives on specific problems with each indicator. These presentations provided an introduction for focused technical work.

Participants engaged in structured discussion of each indicator facilitated by the author to reach consensus on the nature and locus of problems within the metadata, and concrete solutions to address problems identified. Consensus was achieved through plenary discussion documented in real time on a projected screen and agreed by voice vote. A set of recommendations for each indicator was formulated.

## Key recommendations

Problems identified fell into eleven categories. Problem distribution and frequency across all ten indicators is summarized in **Table 3**.

**Table 3:** Summary of problems identified in ten EPMM indicators

Nature or locus of problem	Percent of indicators affected by type (frequency)		Indicator numbers affected		Total percent of indicators affected
**MH Policy**	**MH Finance**	**MH Policy**	**MH Finance**	100%
1. Underlying construct for measurement	100% (5/5)	100% (5/5)	1, 2, 3, 4, 5	6, 7, 8, 9, 10	80%
2. Composition (components of numerator or denominator)	80% (4/5)	80% (4/5)	1, 2, 4, 5	6, 7, 8, 9	70%
3. Method of calculation (scoring)	80% (4/5)	60% (3/5)	1, 2, 4, 5	6, 7, 10	50%
4. Data source(s)	40% (2/5)	100% (5/5)	1, 2	6, 7, 8, 9, 10	60%
5. Operationalized definition of terms	80% (4/5)	40% (2/4)	2, 3, 4, 5	8, 9	60%
6. Disaggregation	60% (3/5)	60% (3/5)	1, 2, 4	6, 8, 10	50%
7. Transparency (availability in the public domain)	40% (2/5)	60% (3/5)	2, 3	6, 9, 10	40%
8. Level of analysis (global, national or subnational)	60% (3/5)	20% (1/5)	1, 2, 3	8	40%
9. Ownership (data custodian)	20% (1/5)	60% (3/5)	3	6, 9, 10	30%
10. Periodicity	40% (2/5)	20% (1/5)	2, 3	9	30%
11. Relevance for maternal health monitoring	20% (1/5)	20% (1/5)	3	6	20%

MH – maternal health, EPMM – Ending Preventable Maternal Mortality

### Consultation 1: Selected Maternal Health Policy Indicators

#### 1) Legal status of abortion

Recommendations:

1. Implement directionality in the value of the indicator based on evidence demonstrating the association between legal grounds and outcomes of interest (eg, safety, access) to allow tracking.
2. Create a scoring hierarchy that progresses from most to least restrictive, using a color coding system.
3. Transform the criteria from national categorical responses (Yes/No) to capture responses disaggregated by sub-national geographies.
4. Develop signal functions for abortion based on guidelines for accessibility, availability, acceptability, and quality (AAAQ) of abortion services in the WHO Global Abortion Policy Database (GAPD) [23].
5. Add sub-measures to allow global comparisons of abortion legality as part of the enabling environment for maternal health:
  - Evidence of elements of AAAQ as defined in the WHO GAPD guidelines
  - Types of provider authorized to provide legal abortion
  - Census of authorized abortion providers

#### 2) Is there a national policy to ensure engagement of civil society organization (CSO) representatives in periodic review of national programs for reproductive maternal newborn child adolescent health (RMNCAH)?

Recommendations:

1. Adjust the indicator to measure engagement directly. However, country representatives endorsed monitoring the existence of a policy requiring CSO representation until direct measurement of effective engagement is feasible.
2. Define engagement of CSO representatives:
  - Incorporate lessons from Ocloo & Matthews (2016) to operationalize the definition of engagement in a meaningful way [24].
  - Define measures and data sources to evidence engagement, eg, registry of public comment periods, minutes reflecting participation in formulation of policy revisions needed, record of interventions, etc.
3. Categorize responses by types of CSOs engaged (eg, international non-governmental organizations [NGOs], local NGOs), rather than by review of some vs. all components of RMNCAH programs. Include the latter as a disaggregation factor.
  - Highlight inclusion of CSOs that represent marginalized populations within the definition of CSOs in the survey instructions. Consider national adaptations designating specified marginalized groups to drive improvement in their representation in those countries.
4. Reaffirm the intent of this indicator is for national-level monitoring. Prioritize engagement of subnational and national CSOs in review of national RMNCAH programs.
  - Specify optimal respondents in the survey instructions (i.e., the data source).
5. Develop a scoring system based on organizational maturity, eg, a five-point scale from nascent to mature, using operational definitions to be included in the indicator.
6. Define “periodic review” as “assessment of progress on indicators in the national RMNCAH strategy” and specify that it must be “participatory”.
  - Define periodicity (should be defined by those leading the RMNCAH programs)
    - Align with the RMNCAH WHO Policy Survey.
    - Align with the related EPMM indicator, number 3), below.
7. Require documentation of the written policy, with evidence of implementation guidelines within the national strategic document, as the data source.
  - Ensure that the source document is appended.
  - Include reports/minutes of the periodic reviews.

#### 3) Presence of a national set of indicators with targets and annual report to inform annual health sector reviews and other planning cycles

Recommendations:

1. Clarify the intended construct for measurement, eg:
  - Governance structure adequate to guide planning and monitoring of health issues and responses
  - Country ownership of selection of indicators and targets for national MNH monitoring
  - Improvement of the mechanism for national MNH monitoring overall
2. Separate the current indicator into three related but separate measures:
  - Existence of national set on indicators/targets
  - Analysis of the data through generation of an annual report
  - Evidence of meaningful use of the information
3. Specify the level of use:
  - For global monitoring, limit the indicator to existence of a national set of indicators with targets.
  - For national/subnational use, include data to measure active monitoring and use of the national set of indicators with targets.
4. Adjust the indicator definition to “Specification of a national set of MNH indicators with annual reporting of current estimates/values, available in the public domain”.
  - Remove “and other planning cycles”.
5. Specify that “current values” should be reported and capture periodicity for each component indicator, as not all indicators are annual.
6. Specify a scoring mechanism, modeled after those proposed by SCORE or MEASURE Evaluation [25,26].

#### 4) Presence of laws and regulations that guarantee women aged 15-49 access to sexual and reproductive health (SRH) care, information, and education

Recommendations:

1. Conduct a systematic review of empirical evidence and/or human rights entitlements to substantiate the construct validity for each component.
2. Remove age limits from the indicator definition; consider any age limits in place a restriction.
3. Disaggregate by state for countries with differing laws and regulations for component parts.
  - Harmonize with SDG 17.18.1.: “Proportion of sustainable development indicators produced at the national level with full disaggregation when relevant to the target, in accordance with the Fundamental Principles of Official Statistics”.
4. Revise the scoring mechanism to address the following specific problems:
  - All components are arbitrarily equally weighted, but their specificity varies greatly (eg, “maternity care” is included as a single component).
  - It is impossible to distinguish between national- and state-level variations.
  - Subtracting barriers from enablers to calculate the indicator score is sensitive to the number of barriers and enablers included.
  - The total score is calculated based on individual components, not section scores (the mean of components within each section). Calculating the total score by taking the average of the individual components across all sections arbitrarily assigns more importance to sections with more components than others, rather than giving all four sections equal weight.

#### 5) Proportion of women aged 15-49 who make their own informed decisions regarding sexual relations, contraceptive use, and reproductive health care

Recommendations:

1. Articulate the construct for measurement clearly, eg:
  - Women’s bodily autonomy and agency over decisions that affect her personally
  - Women’s empowerment within society and/or within her intimate partner relationships
2. Evaluate whether the intended construct encapsulates all three components of this indicator. Conduct validation research to ascertain whether data for all components demonstrate convergent validity.
3. If the evaluation suggests no strong unifying construct, uncouple the components and report them separately.
  - Provide a human rights- and evidence-based analysis of the basis for each component through a systematic review of the literature.
  - Conduct qualitative research to explore social determinants that influence or explain the outcomes of interest.
4. Add supplemental response options to explore root cause factors that limit or influence decision-making, eg, access to financial resources, required 3rd party authorization, etc.
5. Correlate, validate, and harmonize with the SWPER survey-based index for women's empowerment [27], which uses DHS data on decision making to allow comparable measures across time and countries.
6. Adjust the denominator.
  - If the components are uncoupled, there is no need for a common denominator.
  - Remove “currently using contraception” from the denominator, particularly for the question about a woman’s ability to refuse sexual intercourse.
  - Expand the survey and data sources beyond Demographic and Health Surveys (DHS) (eg, Multiple Indicator Cluster Surveys [MICs] or Performance Monitoring for Action [PMA2020]) to address issues identified with the denominator that are dictated by the DHS format.
7. Remove the word “informed” from the definition, or define it operationally for all three components.
8. Revise the scoring module:
  - Report each domain score and the total. Score components separately for each of the three domains, and take the average for each domain (instead of multiplying, which gives a value that is too small and hard to interpret).
  - Make scoring binary for each component as follows:
    - For Questions 1 & 2, collapse and report “Mainly alone” or “Joint decision” (affirmative responses that count toward empowerment) vs. “Mainly husband” or “partner and Other/Specify” (responses that do not count toward empowerment)
    - For Question 3, report “Yes” vs. “Depends/Not Sure”
  - Study and explore systematic differences between those who answer in the affirmative for all three questions vs. those who do not.

### Consultation 2: Selected Maternal Health Financing Indicators

#### 6) Out-of-pocket expenditure as a percentage of total expenditure on health

Recommendations:

1. Address out-of-pocket expenditure on maternal health specifically:
  - Specify standard disaggregation factors, including disaggregation by MNH similar to International Conference on Population and Development (ICPD) global survey [28]; India’s National Family Health Survey [29], PMA2020 [30], DHS, Service Provision Assessments (SPA).
  - Alternatively, create a RMNCH module similar to DHS context-specific modules.
2. Specify data sources. Revise the DHS maternal health module to include questions related to out-of-pocket maternal health expenditure as a percentage of total household expenditure.
3. Advocate to WHO and national governments to make all data sources and full metadata, and not just the final reported indicator value, available in the public domain:
  - Request a public-access data hub at the country government level.
  - Report total government expenditure disaggregated by condition.
  - Make metadata available to allow examination of line item expenditure.
4. Improve and standardize the methodology:
  - Improve survey methodology by implementing standard recall period, optimal number of questions, questions grouped by type of expenditure, and probes to capture non-service related expenditure.
  - Capture the estimated opportunity cost to people who cannot access care because of cost-prohibitions, to make the indicator “pro-poor.”
  - Adjust the denominator to total household expenditure (not total health expenditure) to harmonize with SDG Target 3.8.2 [31], so this indicator is no longer constrained by National Health Account limitations.
  - Disaggregate by funding source, using coding similar to the Organization for Economic Co-operation and Development (OECD) Development Assistance Committee (DAC) and World Health Organization (WHO), which allow disaggregation of donors.
5. Improve reporting:
  - Report both directly-derived country values and data from special surveys not constrained by the national accounting framework (which requires a zero balance) separately, and triangulate to compare validity of these estimates.
  - Regularly report the percentage of out-of-pocket expenditure attributable to maternal health in comparison to the percentage of out-of-pocket expenditure for other disease conditions (these data are available for many countries but are not routinely reported) [32].
6. Ensure intersectoral coordination between data custodians and stewards in the finance and health sectors at global and country levels:
  - The data custodian for this indicator at the global level is the WHO National Health Accounts team in the Health Financing division, and at country level, the National Statistical Offices/National Health Accounts. At global level, ensure ongoing internal coordination with WHO divisions of Sexual and Reproductive Health (SRH) and Maternal Child Adolescent Health (MCA), and at country level with Ministries of Health maternal health divisions to improve this indicator for maternal health monitoring.

#### 7) Are the following (maternal health-related) services provided free of charge at point of use in the public sector for women of reproductive age?

Recommendations:

1. Enumerate specific services in the area of childbirth to reflect lifesaving interventions for complications. Alternatively, define a minimum essential covered services package.
2. Change the estimation method to calculate this indicator by type of service that should be free rather than category of woman who must pay, for the following reasons:
  - Some services are more likely to throw users into catastrophic spending (eg, C-section has greater costs incurred than immunization)
  - This method still allows disaggregation by individual-level equity factors (wealth, age, geography, etc.)
  - Evidence shows that targeting is less effective than universal coverage and has human rights implications.
3. Remove age limits.
4. Change the data source to use primary data collected via household or facility survey from women on any charges, formal or informal, that they have paid for care.

#### 8) Costed implementation plan for maternal, newborn, and child health (MNCH)

Recommendations:

1. Clarify the underlying construct for measurement: national governance capacity to develop, cost, execute, and review a plan for MNCH.
2. Develop additional questions and analysis to strengthen the indicator’s ability to capture the intended construct:
  - Start with the following categorical question: “Is there a stand-alone costed national plan for MNCH (that is not just part of a larger health strategy)?”
  - Include further probes to determine the quality of the costing exercise (eg, does it include current/capital costs?)
  - Given the trend toward decentralized health systems, measure the national government’s function to harmonize:
    - across accounts
    - costed plans from subnational level
    - different financing sources (private sector, debt funding, donor funding)
3. Develop a tool to systematically assess the adequacy of the costing exercise and data sources submitted, to explore national costing capacity.
4. Expand the definition of a “national implementation plan” to include subnational plans, if these are the basis for planning and accounting.
  - Add a discriminating question first, to determine whether the country is a federal state with decentralized planning (“Yes”/”No”).
  - Measure the proportion of funding for the Consumer Price Index (CPI) that is budgeted at subnational level.
  - Systematically analyze national governance in federal/decentralized states, as well as coordination of plans and budgets between the Ministries of Health and Finance. Collect evidence of effective coordination.
5. Evaluate the response rate and effectiveness of the survey questions through cognitive interviews, item analysis, etc. and implement changes to improve survey quality.

#### 9) Annual reviews are conducted of health spending from all financial sources, including RMNCH spending, as part of broader health sector reviews

Recommendations:

1. Clarify the construct for measurement. Specify that the outcome of interest is occurrence of a routine “broad health sector review”, and the factor tracked is whether it includes review of health spending from all sources by condition (including RMNCH).
2. Define “broad health sector review” and specify the inputs that should be included for review.
3. Determine the optimal frequency for the review, given the burden and the periodicity for updates to the data that are included.
4. Specify that “all financial sources” include both government and external sources.
5. Adjust the question as follows:
  “Is there a national health sector review?” (Yes/No)
    - If Yes, “How often? When was the last one?”
    - “Does it include review of health spending? If so, from which sources (enumerate financial sources that should be included)?”
    - “Does it review spending by condition? If so, does that include RMNCH?”
    - Specify that documents must be appended.
6. Identify a data custodian for this indicator, eg,:
  - WHO RMNCAH Policy Survey
  - Partnership for MNCH (PMNCH) Health Financing Group
  - Consult the Independent Accountability Panel, as this indicator was adapted from a recommendation made by its predecessor, (Commission on Information and Accountability) CoIA.

#### 10) Percentage of total health expenditure spent on reproductive, maternal, newborn, and child health

Recommendations:

1. To emphasize this indicator’s focus on accountability:
  - Revise the numerator to focus on government sources only. (Note: this will exclude the majority of the budget, which comes from ODA, in many countries). Alternatively, disaggregate by source.
  - Adjust the denominator to government expenditure instead of all sources.
2. Report absolute expenditure by condition rather than the percentage of total expenditure. A relative measure risks pitting conditions against each other.
3. Disaggregate government spending versus ODA/other spending to help push governments toward self-sufficiency in the area of health where there is disproportionate reliance on ODA.
4. Demand transparency of data sources (national budgets), to allow CSOs and other stakeholders to review and calculate the disaggregated data on spending by condition.
5. Conduct validation research to explore the relative validity of similar indicators measuring this construct, e.g. “Current country health expenditure per capita (including specifically on RMNCAH) financed from domestic sources” [33]. Harmonize the indicator reported by global initiatives working to improve tracking of ODA and domestic health financing based on the results.
6. Identify an appropriate data custodian for this indicator, eg;
  - GFF
  - WHO Global Action Plan and partners [34]
  - CSO budget accountability organizations [35,36]

## DISCUSSION AND CONCLUSIONS

This paper summarizes weaknesses encountered with ten global maternal health indicators prioritized for monitoring progress toward ending preventable maternal mortality and proposes specific solutions to strengthen them. Eleven types of problems were identified, about which some generalizations can be made. The recommended solutions are, for the most part, specific to each indicator.

Of note, lack of clarity and conceptual precision in the underlying construct for measurement was identified in all ten indicators and, thus, construct validity was suboptimal for all indicators reviewed. Similarly, a majority of indicators exhibited issues with components in the numerator or denominator, and lacked operationalized definitions for key terms. Benova and colleagues [37] highlight the primary importance of theoretical clarity about the concept intended for measurement, including its intended purpose, meaningfulness, and utility, in their scoping review and definitional framework of indicator validity. Construct validity is overarching and subsumes other types of validity, since accurate measurement of a poorly operationalized or irrelevant concept will still lack validity. Furthermore, poor operationalization of the construct into the components of the numerator and denominator, or of specific terms therein, are further threats to validity.

For a majority of indicators, data sources were not standard, not validated, or not available in the public domain. These findings underscore calls for greater data transparency to build trust in global health measures [38], and in fiscal governance for health [39] by scholars and advocates who highlight that indicator data sources should be available in the public domain to allow stakeholders to replicate, verify, and improve indicators of importance in their context. In maternal health financing, lack of transparent data are further compounded by lack of disaggregation by condition, making it especially difficult to track adequate budget allocation, actual spending, and out-of-pocket expenditure on maternal health specifically.

A problem intrinsic to many maternal health policy indicators is that they document the presence of a policy rather than its performance upon implementation. Without defined targets, values, directionality, or a scoring mechanism to measure trends, it is difficult to use them to track change. Issues with the methods for estimation were identified in 4/5 of maternal health policy indicators and 3/5 of finance indicators.

Our consultation process produced concrete recommendations to strengthen indicators identified as among the best available measures for tracking progress toward priority recommendations in the EPMM Strategies. A strength of this process was that it included representatives of the data custodian agencies for indicators under discussion, as well as academic and programmatic experts from a range of country contexts. A limitation is that expert opinion from non-systematic review is a relatively weak level of evidence [40]. However, these recommendations are grounded in a high level of collective experience and specific expertise from diverse sources.

These recommendations if implemented can improve the construct validity, reliability, and data quality of important indicators for ending preventable maternal mortality. Further validation research is needed for many maternal health indicators in use, especially at health policy and governance levels, including those covered in this review.

## Additional material


Online Supplementary Document
