# Supplementary material for: Recommendations for refining key maternal health policy and finance indicators to strengthen a framework for monitoring the Strategies toward Ending Preventable Maternal Mortality (EPMM)
Source: J Glob Health. 2021 Oct 23;11:02004. doi: 10.7189/jogh.11.02004 (PMC8561338; doi:10.7189/jogh.11.02004)
Supplement: Online Supplementary Document [file jogh-11-02004-s001.pdf]

Improving Maternal Health Measurement Capacity and Use (IMHM)
Consultation to Strengthen Five Maternal Health Policy Indicators
DECEMBER 2018 | London, UK

PARTICIPANTS AND SPEAKERS- CONSULTATION 1

Richard Adanu
School of Public Health
University of Ghana
Legon, Ghana

Tariq Azim
Speaker: Indicator 3
MEASURE Evaluation
Washington, DC, USA

Sara Bandali
Speaker: Indicator 2
Evidence for Action
London, England, UK

Eduardo Bergel
Institute for Clinical Effectiveness and Health Policy (IECS)
Rosario, Argentina

Nyasha Chingore-Munazvo
(not present)
The Center for Reproductive Rights
Nairobi, Kenya

Elahi Chowdhury
icddr,b
Dhaka, Bangladesh

Fernanda Ewerling
Federal University of Pelotas
Pelotas, Brazil

Veronique Filippi
The London School of Hygiene and Tropical Medicine
London, England, UK

Alfredo Fort
Speaker: Indicators 4, 5
UNFPA
New York, NY, USA

Lynn Freedman
Columbia Mailman School of Public Health
New York, NY, USA

Aminu Garba
Speaker: Indicator 2
African Health Budget Network
Abuja, Nigeria

Veloshnee Govender
World Health Organization
Geneva, Switzerland

Zelee Hill
The London School of Hygiene and Tropical Medicine
London, England, UK

Eleanor Hukin
Options UK
London, England, UK

Ronnie Johnson
Speaker: Indicator 1
World Health Organization
Geneva, Switzerland

Rima Jolivet
Women & Health Initiative,
Harvard T.H.Chan School of Public Health
Boston, MA, USA

Lovney Kanguru
The University of Aberdeen
Aberdeen, Scotland, UK

Sunita Kishor
Speaker: Indicator 5
ICF International
Rockville, MD, USA

Tiziana Leone
(not present)
Sent Slides: Indicator 1
The London School of Economics and

Zoe Matthews
University of Southampton
Southampton, UK

Allisyn Moran
Speaker: Indicators 2, 3
World Health Organization Geneva,
Switzerland

Sara Nam
Options UK
London, England, UK

Felix Ibrahim Obi
Speaker: Indicator 3
African Health Economics Association
Abuja, Nigeria

Tom Pullum
Speaker: Indicator 5
ICF International/DHS
Rockville, MD, USA

Sowmya Ramesh
Population Council
New Delhi, India

Jennifer Requejo
UNICEF
New York, NY, USA

Sarah Smiley
Women & Health Initiative,
Harvard T.H. Chan School of Public
Health
Boston, MA, USA

Laura Sochas
Speaker: Indicator 1
The London School of Economics and
Political Science
London, England, UK

Olivia Tulloch
Options UK
London, England, UK

Kavitha Viswanathan
(not present)
Sent Slides: Indicator 3
World Health Organization
Geneva, Switzerland

Charlotte Warren
Population Council
Washington, DC, USA

Improving Maternal Health Measurement Capacity and Use (IMHM)
Consultation to Strengthen Five Maternal Health Financing Indicators
DECEMBER 2018 | London, UK

PARTICIPANTS AND SPEAKERS- CONSULTATION 2

Richard Adanu
School of Public Health
University of Ghana
Legon, Ghana

Bilal Avan
The London School of Hygiene and
Tropical Medicine
London, England, UK

Eduardo Bergel
Institute for Clinical Effectiveness
and Health Policy (IECS)
Rosario, Argentina

Stephan Brenner
Speaker: Indicator 7
University of Heidelberg
Heidelberg, Germany

Oona Campbell
(not present)
The London School of Hygiene and
Tropical Medicine
London, England, UK

Elahi Chowdhury
icddr,b
Dhaka, Bangladesh

Aminu Garba
Speaker: Indicators 7, 8, 9
African Health Budget Network
Abuja, Nigeria

Veloshnee Govender
Speaker: Indicator 7
World Health Organization
Geneva, Switzerland

Giulia Greco
The London School of Hygiene and
Tropical Medicine
London, England, UK

Karen Hoehn
Independent Consultant
Brussels, Belgium

Rima Jolivet
Women & Health Initiative,
Harvard T.H.Chan School of Public Health
Boston, MA, USA

Grace Kabaniha
Speaker: Indicator 6
World Health Organization
Congo, Brazzaville

Tiziana Leone
The London School of
Economics and Political Science
London, England, UK

Zoe Matthews
University of Southampton
Southampton, UK

Ann-Beth Moller
Speaker: Indicator 9
World Health Organization
Geneva, Switzerland

Allisyn Moran
Speaker: Indicator 8
World Health Organization
Geneva, Switzerland

Sarah Neal
University of Southampton
Southampton, England, UK

Felix Ibrahim Obi
Speaker: Indicator 10
African Health Economics Association
Abuja, Nigeria

Sowmya Ramesh
Population Council
New Delhi, India

Jennifer Requejo
Speaker: Indicator 10
UNICEF
New York, NY, USA

Alice Sabino
Options UK
London, England, UK

Neha Singh
The London School of Hygiene and
Tropical Medicine
London, England, UK

Sarah Smiley
Women & Health Initiative,
Harvard T.H. Chan School of Public Health
Boston, MA, USA

Laura Sochas
Speaker: Indicator 6
The London School of Economics and
Political Science
London, England, UK

Kavitha Viswanathan
(not present)
Sent Slides: Indicator 9
World Health Organization
Geneva, Switzerland

Charlotte Warren
Population Council
Washington, DC, USA
